# A Administração Oral de Nitrito a Curto Prazo Diminui a Rigidez Arterial em Ratos Wistar Treinados e Sedentários

**DOI:** 10.36660/abc.20230783

**Published:** 2024-11-25

**Authors:** Thiago Pereira Souza, Lidieli Pazin Tardelli, Rafael Antunes Nicoletti, André Mourão Jacomini, Gabriel Francisco de Mello Martins, Lucas Cézar Pinheiro, José Eduardo Tanus-Santos, Sandra Lia do Amaral, Anderson Saranz Zago

**Affiliations:** 1 Universidade Estadual Paulista Departamento de Educação Física Bauru SP Brasil Universidade Estadual Paulista (UNESP) - Departamento de Educação Física, Bauru, SP – Brasil; 2 Universidade Federal de São Carlos Programa Interinstitucional de Pós-Graduação em Ciências Fisiológicas São Carlos SP Brasil Universidade Federal de São Carlos (UFSCar) - Programa Interinstitucional de Pós-Graduação em Ciências Fisiológicas, PIPGCF UFSCar/UNESP, São Carlos, SP – Brasil; 3 Universidade Federal de Santa Catarina Departamento de Farmacologia Florianópolis SC Brasil Universidade Federal de Santa Catarina (UFSC) - Departamento de Farmacologia, Florianópolis, SC – Brasil; 4 Universidade de São Paulo Departamento de Farmacologia Ribeirão Preto SP Brasil Universidade de São Paulo - Departamento de Farmacologia, Ribeirão Preto, SP – Brasil

**Keywords:** Óxido Nítrico, Nitritos, Exercício Físico, Análise de Onda de Pulso, Hipertensão

## Abstract

**Fundamento:**

O óxido nítrico (NO) desempenha um papel importante na regulação da pressão arterial (PA), atuando diretamente na resistência vascular periférica por meio da vasodilatação. O treinamento físico (via eNOS/NO) e a ingestão de nitrito foram considerados os principais estímulos para o aumento do NO.

**Objetivo:**

No presente estudo, examinamos os efeitos da administração oral de nitrito e do treinamento com exercícios aeróbicos sobre a PA e rigidez arterial de ratos Wistar.

**Métodos:**

Trinta e nove (39) ratos Wistar machos jovens foram divididos nos seguintes grupos (n=9 ou 10 por grupo): Sedentário-Controle (SC), Sedentário-Nitrito (SN), Treinado-Controle (TC) e Treinado-Nitrito (TN). Eles foram submetidos a treinamento físico aeróbico em esteiras por 8 semanas (50-60% da capacidade física, 1h/dia, 5 dias/semana) ou mantidos sedentários. Nos últimos 6 dias de treinamento, nitrito foi administrado oralmente (15 mg/Kg por gavagem). A PA, a rigidez arterial e as concentrações plasmáticas e teciduais de nitrito foram avaliadas após o treinamento e o período de administração oral de nitrito. O nível de significância foi definido como p < 0,05.

**Resultados:**

A administração oral de nitrito foi eficaz na redução dos valores de rigidez arterial (TN, -23%; e SN, -15%). Ambos os grupos que tiveram apenas um tipo de intervenção apresentaram PA sistólica menor em comparação com o controle (TC vs. SC, -14,23; e SN vs. SC, - 12,46).

**Conclusão:**

Assim, concluímos que a administração oral de curta duração (6 dias), associada a um programa de treinamento físico aeróbico promovem diversos benefícios hemodinâmicos em ratos Wistar machos, como melhorias na rigidez arterial e na PA. Essas respostas sugerem que o treinamento físico e a suplementação de nitrito de sódio podem ser alternativas satisfatórias para a prevenção e tratamento da hipertensão.

## Graphical abstract

**Figura Central:**
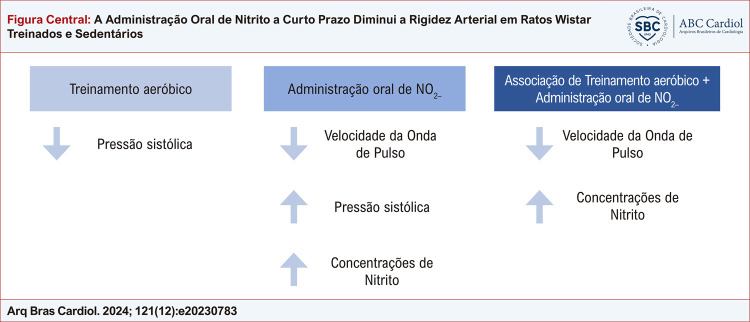
: A Administração Oral de Nitrito a Curto Prazo Diminui a Rigidez Arterial em Ratos Wistar Treinados e Sedentários

## Introdução

A literatura científica afirma que a pressão arterial (PA) deve estar próxima de 110-115 mmHg para pressão arterial sistólica (PAS) e 70-75 mmHg para pressão arterial diastólica em indivíduos saudáveis. Valores acima destes estão relacionados a uma alta incidência de eventos cardiovasculares e mortalidade, como acidente vascular encefálico, infarto agudo do miocárdio, entre outros.^1-4^ Múltiplos fatores estão envolvidos na regulação da PA, incluindo a rigidez arterial, que é caracterizada pela perda de complacência das paredes arteriais.^5,6^ Atualmente, devido às suas consequências, ela tem sido associada como um preditor de risco de mortalidade cardiovascular, mesmo na população em geral,^7,8^ uma vez que artérias mais rígidas promovem um suprimento sanguíneo menos eficiente através dos vasos para os tecidos e órgãos, colocando demandas crescentes sobre o coração.^9^

Por outro lado, o treinamento físico é recomendado para neutralizar esses efeitos, prevenindo a HT, diminuindo o tônus simpático, melhorando a resistência vascular periférica e a rigidez arterial.^10-12^ O exercício físico é capaz de melhorar muitos mecanismos relacionados à regulação dos níveis de PA, como a produção de óxido nítrico (NO) e sua biodisponibilidade.^13-15^

Considerado um potente vasodilatador, o NO pode ser produzido por meio de duas vias metabólicas principais: a) via eNOS/NO, a produção de NO via eNOS ocorre a partir da clivagem da L-arginina em NO e L-citrulina. No treinamento de exercícios aeróbicos, isso ocorre por meio de estimulação física devido ao aumento do débito cardíaco e consequente promoção de estresse de cisalhamento sobre as células endoteliais vasculares.^16^ Essa via recebe grande influência do treinamento físico;^17,18^ e b) Via Nitrato/Nitrito/NO, na qual a concentração de NO é aumentada por meio da administração oral de nitrito (NO_2-_) e nitrato (NO_3-_).^19,20^ Inicialmente, o nitrito ingerido é convertido em NO sob as condições ácidas do estômago, enquanto o restante entra na circulação sanguínea. Nos tecidos, o nitrito restante é ainda mais reduzido sob as condições de pH baixo e hipóxia por proteínas redutase, convertendo nitrito em NO, sendo considerado uma boa fonte de NO independente da eNOS.^21-23^

Conforme mencionado anteriormente, a prática de exercício físico e a ingestão de nitrato e nitrito são considerados grandes estímulos para o aumento da concentração de NO. No geral, independentemente da fonte de produção (vias eNOS/NO/GMPc ou Nitrato/Nitrito/NO), o NO desempenha um papel importante no controle da PA, atuando diretamente sobre a resistência vascular periférica por meio da vasodilatação.^24^ Entretanto, ainda não está claro quais seriam os efeitos da combinação das duas intervenções no aumento da concentração de NO e seu impacto sobre a rigidez arterial.

Portanto, o objetivo deste estudo foi verificar os efeitos da administração oral de nitrito e do treinamento com exercícios aeróbicos sobre a PA e a rigidez arterial de ratos Wistar. No presente estudo, levantamos a hipótese de que a administração oral de nitrito, associada ao treinamento físico, promove efeitos adicionais na regulação da PA e na rigidez arterial do que intervenções isoladas.

## Métodos

### Animais

Quarenta ratos Wistar (8 semanas de idade/machos/250-300g) foram obtidos do Biotério do Hospital das Clínicas da Faculdade Estadual de Medicina de São Paulo (UNESP-Botucatu, SP, Brasil). Os animais foram alojados no Biotério da UNESP-Faculdade de Ciências (Bauru, SP, Brasil), em gaiolas de até cinco animais, em ciclo claro/escuro de 12 horas e temperatura controlada (22 °C). Todos os procedimentos foram aprovados pelo Comitê de Ética para Uso de Animais (CEUA) da Universidade Estadual Paulista, Bauru, Brasil (protocolo n.º 003/2018).

### Grupos experimentais, treinamento físico e tratamento farmacológico

Inicialmente, todos os animais passaram por um período de adaptação de 10 dias em esteira rolante (5-10 m/min, 5 minutos), sendo submetidos ao teste de capacidade máxima (Tmáx), que consiste em correr em esteira rolante progressiva com incrementos de 5 m/min a cada 3 minutos, até a exaustão, conforme descrito anteriormente. O Tmáx foi repetido no final da 4ª semana, com o objetivo de ajustar a velocidade para manter a intensidade do treinamento, e ao final do protocolo experimental, para verificar os efeitos do treinamento na capacidade física.^25,26^Após os testes iniciais, todos os ratos foram separados em quatro grupos com peso corporal, velocidade da onda de pulso e valores basais de desempenho do Tmáx semelhantes. Então, esses grupos foram selecionados aleatoriamente para compor os seguintes grupos (n = 9 ou 10 por grupo): (1) Sedentário-Controle (SC), (2) Sedentário-Nitrito (SN), (3) Treinado-Controle (TC) e (4) Treinado-Nitrito (TN). O método de randomização escolhido foi a randomização simples. O tamanho da amostra foi baseado em estudos anteriores presentes.^27,28^ Os grupos de treinamento realizaram treinamento físico aeróbico em esteira ergométrica (Inbramed, Millenium, Brasil), com intensidade moderada, a 50-60% do Tmáx, durante 1h/dia, 5 dias por semana, por 8 semanas, enquanto os grupos sem exercícios exercitados permaneceram sedentários. Nos últimos 6 dias de treinamento, o nitrito foi administrado.

Nitrito de sódio (15 mg/kg de peso corporal - Dinâmica®) ou veículo (água) foram administrados por gavagem uma hora antes do treinamento físico (às 9h). A concentração de nitrito de sódio foi determinada em conformidade com a literatura, que demonstra os efeitos anti-hipertensivos e antioxidantes do composto.^29-31^

### Velocidade da onda de pulso (VOP)

O método da velocidade da onda de pulso (VOP) tem sido amplamente utilizado para a avaliação da rigidez arterial, sendo considerado o padrão ouro para a complacência arterial.^32,33^

Para avaliar a VOP, os animais foram anestesiados com cloridrato de cetamina e xilazina (50 mg/kg e 10 mg/kg, respectivamente, IP) e colocados em decúbito ventral sobre uma mesa aquecida. Dois sensores pOpet ® (Axelife SAS, Saint Nicolas de Redon, França) foram colocados no membro anterior e posterior direito. A distância percorrida (D, m) estimou a distância entre as duas sondas e o tempo de trânsito (TT, ms), medido pelo software pOpet 1.0, foram usados para calcular a VOP pela seguinte fórmula: VOP(m/s) = D(m)/TT(s). Este método teve como objetivo avaliar a rigidez arterial, tendo recentemente demonstrado boa validação.^32^ A média de dez medições foi considerada como resultado. Esta avaliação ocorreu em dois momentos, no início do experimento e ao final do período de treinamento físico e tratamento.

### Avaliação não invasiva da pressão arterial

O sistema de pletismografia de manguito de cauda foi usado para determinar a PA indireta (PanLab LE5001, Barcelona, Espanha). Todos os animais foram adaptados por 5 dias ao tubo cilíndrico de acrílico, que os manteve em repouso. Os animais foram mantidos em tubo cilíndrico de acrílico, pré-aquecido (37 °C), para promover a vasodilatação da artéria caudal, e então aguardaram por 5-10 minutos para garantir que estivessem em repouso. O manguito foi colocado na porção proximal da cauda e conectado ao esfigmomanômetro para inflar. A medida foi analisada pelo transdutor de pressão (IITC Inc. Life Science - MRBP-r). A avaliação ocorreu 1 hora após a última ingestão de nitrito e a média de cinco medidas (intervalo de 1 minuto entre elas) foi considerada como resultado.^33^

### Procedimentos de eutanásia

Os animais foram eutanasiados por excesso de anestésicos, com cloridrato de xilazina (ANASEDAN®, 40mg/kg) e cloridrato de cetamina (DOPALEN®, 160mg/kg), VETEBRANDS Brasil (proporção 1:1, 0,1mg/100g de peso corporal), e então, decapitados. A eutanásia ocorreu 2 horas após a última ingestão de nitrito. Plasma, músculo cardíaco, tibial anterior e músculo sóleo foram coletados dos animais.

### Medição das concentrações de nitrito no plasma e tecidos

O plasma, o tecido cardíaco e muscular (~125 mg) foram homogeneizados em um tampão de fosfato. Após a centrifugação, a solução contendo os tecidos ou alíquotas de plasma foi analisada em duplicata para concentrações de nitrito usando o método de quimioluminescência redutiva baseado em ozônio e posteriormente avaliada por um analisador de NO de quimioluminescência em fase gasosa (analisador Sievers Modelo 280 NO; Boulder, CO, EUA), conforme descrito anteriormente.^20,34^

### Análise estatística

Estatísticas descritivas são apresentadas como média ± desvio padrão (DP). O teste de Shapiro-Wilk foi usado para testar a distribuição normal dos dados. Uma ANOVA bidirecional foi usada para identificar diferenças estatísticas na PAS e nas concentrações plasmáticas, musculares e cardíacas de nitrito entre os grupos (SC, SN, TC e TN). Na presença de interações, foi utilizado o teste post hoc de Tukey, e o nível significativo foi definido como p < 0,05.

Para comparação PRÉ e PÓS na VOP dentro dos grupos (SC, SN, TC e TN), uma ANOVA de medidas repetidas foi adotada. Os dados foram analisados usando o pacote estatístico SigmaPlot 12.0 (Systac Software, Inc., San Jose, CA, EUA).

## Resultados

**Figura 1:**
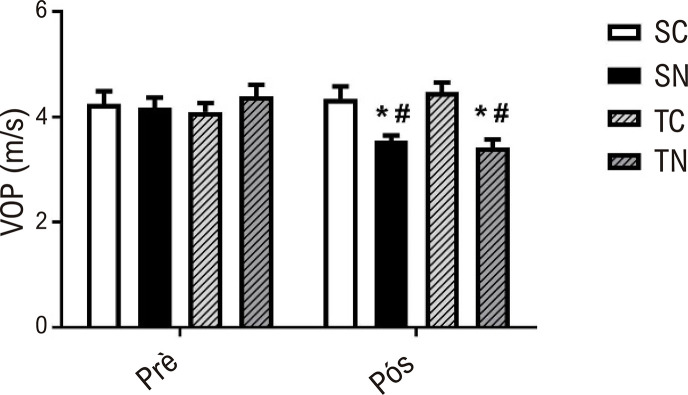
– Velocidade da onda de pulso (VOP) antes (pré) e depois (pós), em SC (Sedentário-Controle, n=10), SN (Sedentário-Nitrito, n=10), TC (Treinado-Controle, n=10) e TN (Treinado-Nitrito, n=9). *Diferenças estatísticas vs. controle respectivo e - (SN vs. SC, p=0,003; e TN vs. TC, p=<0,001); # diferenças estatísticas entre PRÉ e PÓS-intervenção – (TN, p=0,007; SN, 0,011); nível de significância p<0,05.

Na imagem central, apresentamos os principais resultados do nosso estudo. Todos os grupos apresentaram resultados semelhantes para VOP no início do estudo, demonstrando a homogeneidade dos grupos nesse aspecto. Ambos os grupos tratados apresentaram valores de VOP mais baixos em comparação com os grupos não tratados (-19% SN vs. SC; -24% TN vs. TC). A Figura 1 também mostra a comparação entre os momentos pré e pós-intervenção. Não foram encontradas diferenças significativas para os grupos SC e TC para VOP. No entanto, os grupos TN (-23%) e SN (-15%) apresentaram reduções significativas na VOP após o período de treinamento ou tratamento.

**Figura 2:**
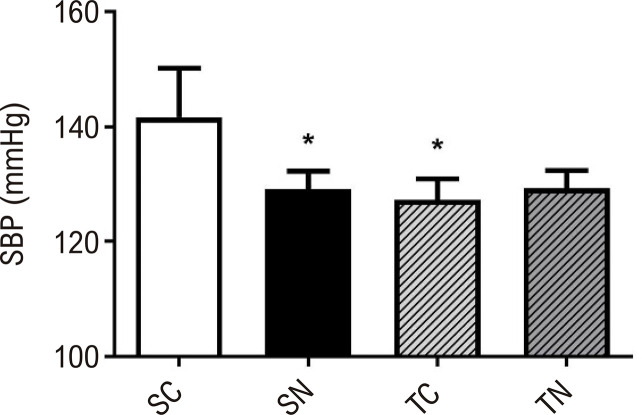
– Valores de pressão arterial sistólica (PAS) em SC (Sedentário-Controle, n=10), SN (Sedentário-Nitrito, n=10), TC (Treinado-Controle, n=10) e TN (Treinado-Nitrito, n=9). *Diferenças estatísticas, TC vs. SC, p=<0,001; SN vs. SC, p=<0,001; nível de significância p<0,05.

A Figura 2 apresenta menor PAS em SN e TC em comparação com o grupo SC. Essa diferença chegou a 12,46 mmHg para o grupo SN (p=<0,001) e 14,23 mmHg para o grupo TC (p=<0,001). No entanto, a combinação de treinamento físico e administração oral de nitrito não apresentou benefícios adicionais àqueles encontrados isoladamente.

**Figura 3:**
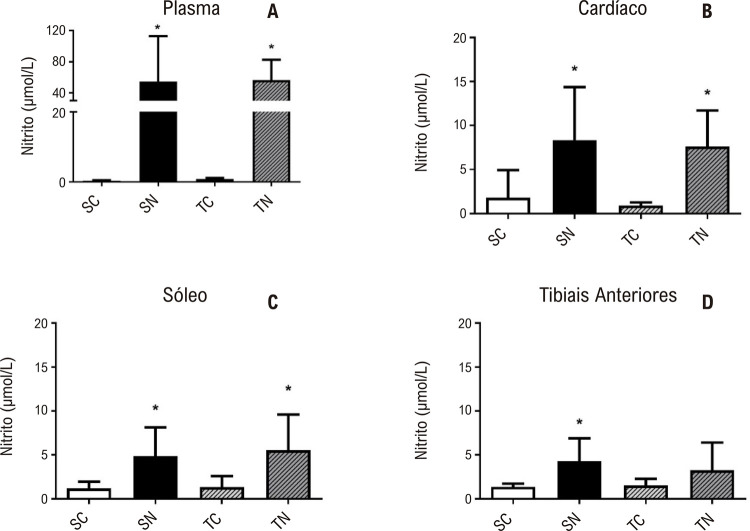
– Concentrações de nitrito no plasma nitrito (A), coração (B), músculo sóleo (C) e músculo esquelético tibial anterior (D) em SC (Sedentário-Controle, n=10), SN (Sedentário-Nitrito, n=10), TC (Treinado-Controle, n=10) e TN (Treinado-Nitrito, n=9). *Diferenças estatísticas, Plasma – SN vs. SC, p=0,006;TN vs. TC, p=<0,001; Coração – SN vs. SC, p=0,002; TN vs. TC, p=<0,001; Sóleo – SN vs. SC, p=0,005; TN vs. TC, p=0,003; Tibial Anterior – SN vs. SC, p=0,019; nível de significância p<0,05.

A Figura 3 apresenta as concentrações de nitrito nos diferentes tecidos. É possível observar que ambos os grupos tratados (SN e TN) apresentaram valores maiores em comparação com os grupos não tratados (SC e TC) no plasma (Figura 3A), coração (Figura 3B) e músculo sóleo (Figura 3C), exceto para o músculo tibial anterior (Figura 3D).

Não foi encontrada diferença estatística entre os grupos SC e TC para a concentração plasmática de nitrito. No entanto, o tratamento com nitrito induziu um aumento no grupo SN (p=0,006) e no grupo TN (p=<0,001) em comparação com seus respectivos grupos de controle.

A magnitude do aumento na concentração de nitrito no músculo cardíaco e esquelético (sóleo) foi menor em comparação com a concentração plasmática de nitrito, no entanto, a mesma tendência foi encontrada. Não foi encontrada diferença entre os grupos SC e TC para concentração cardíaca de nitrito. No entanto, o tratamento com nitrito induziu um aumento no grupo SN (aproximadamente quatro vezes) e no grupo TN (aproximadamente oito vezes) em comparação com seus respectivos grupos de controle.

Para o tecido muscular, nenhuma diferença foi encontrada entre os grupos SC e TC na concentração de nitrito do sóleo e na concentração de nitrito dos tibiais anteriores. Além disso, os grupos tratados (SN e TN) mostraram diferenças em relação aos seus respectivos controles no músculo sóleo (ambos de aproximadamente quatro vezes). No entanto, no músculo tibial anterior, apenas o grupo SN mostrou uma diferença em comparação com o seu controle (aproximadamente três vezes).

## Discussão

O objetivo deste estudo foi verificar o efeito da administração oral de nitrito sobre a PA e a rigidez arterial em ratos treinados e sedentários. Resumidamente, nossos resultados demonstraram que a administração oral de nitrito por um curto período (6 dias) foi relacionada a uma redução de 15% e 22,1% na VOP, nos grupos SN e TN, respectivamente, em comparação com os grupos que não receberam tratamento farmacológico.

A rigidez arterial tem sido extensivamente estudada em modelos animais experimentais,^5,6,32,35^ sendo considerada um importante preditor de risco cardiovascular na população em geral.^7^ Embora o grupo que realizou apenas treinamento físico não tenha apresentado redução da VOP, existem evidências na literatura de que o exercício físico promove benefícios sobre a rigidez arterial. Em uma revisão sistemática de Lopes et al.,^35^ foi demonstrado que o treinamento aeróbico, o treinamento combinado e o treinamento resistido podem promover redução da VOP em pacientes hipertensos.

A concentração de nitrito foi maior em ambos os grupos que receberam suplementação oral de nitrito, um resultado esperado devido ao desenho do estudo. No entanto, esses resultados podem contribuir para melhorias no endotélio vascular e para melhorias na biodisponibilidade de NO. Como consequência, contribui para os efeitos anti-hipertensivos.^18^ Foi demonstrado que a suplementação com nitrito protege o endotélio vascular da atividade antioxidante, reduzindo/inibindo a atividade da NADPH oxidase e da xantina oxidorredutase (XOR) em animais,^36,37^ o que não foi avaliado por este estudo. A inibição da atividade da NADPH oxidase é um mecanismo importante, pois trata-se da fonte mais proeminente de espécies reativas de oxigênio (ERO) e funciona age por meio da inativação do NO.^38-40^ Ling et al.^18^ também demonstraram que a administração oral de nitrito promove mudanças positivas na expressão da NADPH oxidase.

Além disso, a administração oral de nitrito também se mostrou eficaz no aumento das concentrações de nitrito no plasma e em outros tecidos (músculo cardíaco e sóleo), com valores mais altos encontrados nos grupos tratados em comparação com os grupos controle. Esses resultados estão de acordo com a literatura, que relata que a suplementação de nitrito aumenta as concentrações de nitrito no plasma e em outros tecidos.^18,30,41^ Por outro lado, no músculo tibial anterior, apenas o grupo sedentário que recebeu tratamento apresentou altos valores de concentração de nitrito. Ressalta-se ainda que a literatura tem demonstrado alterações nas concentrações plasmáticas e teciduais de nitrito ao longo do tempo após a administração oral.^20^ Pinheiro et al. observaram que o aumento das concentrações de nitrito no plasma iniciou-se 15 minutos após o tratamento, permanecendo por até 4 horas; e no músculo cardíaco e esquelético, as concentrações permaneceram altas até 2 horas após o tratamento, demonstrando assim a relação entre concentração e tempo de tratamento. Sendo assim, essa relação confirma nossa hipótese de que a administração oral de nitrito de sódio pode ser uma alternativa satisfatória para aumentar as concentrações de nitrito e, consequentemente, possivelmente a biodisponibilidade de NO.

De acordo com os resultados da PA, ambas as intervenções realizadas isoladamente, seja o treinamento físico ou farmacológico, apresentaram redução da PAS em comparação com o grupo sedentário e não tratado. Embora muitos estudos tenham demonstrado que o treinamento de exercícios aeróbicos não reduz os níveis de PA em animais Wistar.^42,43^ Em nosso estudo, embora os animais utilizados neste estudo tenham sido considerados normotensos (a linhagem dos animais era normotensa), é possível observar que os valores de PAS estavam em torno de 140 mmHg no grupo SC, o que apresentou valores de PA alterados. Sendo assim, o tratamento com nitrito ou treinamento físico foram suficientes para normalizar os valores de PA. Uma possível explicação para esses resultados é o aumento da produção e concentração de NO, a diminuição da concentração de EROs, o aumento do fator de crescimento endotelial vascular, que são responsáveis pela ativação do mecanismo de angiogênese, redução da produção de Ang-II e menor atividade simpática.^10,42,44-46^ Além disso, a suplementação de nitrito demonstrou benefícios no relaxamento vascular,^47^ e melhorias na função e remodelação cardíaca.^48^

As limitações deste estudo incluem o curto período de tratamento com nitrito, o que requer mais estudos para verificar a resposta a longo prazo. Os níveis de PAS dos animais estavam próximos de 140 mmHg, um valor considerado alterado para animais Wistar e, ainda assim, só conseguimos avaliar a PA no final do protocolo. Sendo assim, não podemos afirmar que o treinamento físico e o tratamento reduziram a PAS ao longo do programa.

## Conclusão

Em resumo, a administração oral de curta duração (6 dias) ou um programa de treinamento físico aeróbico promovem diversos benefícios hemodinâmicos em ratos Wistar machos, como melhorias na rigidez arterial e na PA. Essas respostas sugerem que o treinamento físico e a suplementação de nitrito de sódio podem ser alternativas satisfatórias para a prevenção e tratamento da hipertensão.
