# Supplementary material for: A Administração Oral de Nitrito a Curto Prazo Diminui a Rigidez Arterial em Ratos Wistar Treinados e Sedentários
Source: Arq Bras Cardiol. 2024 Nov 25;121(12):e20230783. [Article in Portuguese] doi: 10.36660/abc.20230783 (PMC11634307; doi:10.36660/abc.20230783)
Supplement: Translation

# Translation: Short-term Oral Nitrite Administration Decreases Arterial Stiffness in Both Trained and Sedentary Wistar Rats

**DOI:** 10.36660/abc.20230783i

**Authors:** Thiago Pereira Souza, Lidieli Pazin Tardelli, Rafael Antunes Nicoletti, André Mourão Jacomini, Gabriel Francisco de Mello Martins, Lucas Cézar Pinheiro, José Eduardo Tanus-Santos, Sandra Lia do Amaral, Anderson Saranz Zago

**Affiliations:** 1 Brazil Universidade Estadual Paulista (UNESP) - Departamento de Educação Física, Bauru, SP – Brazil; 2 Brazil Universidade Federal de São Carlos (UFSCar) - Programa Interinstitucional de Pós-Graduação em Ciências Fisiológicas, PIPGCF UFSCar/UNESP, São Carlos, SP – Brazil; 3 Brazil Universidade Federal de Santa Catarina (UFSC) - Departamento de Farmacologia, Florianópolis, SC – Brazil; 4 Brazil Universidade de São Paulo - Departamento de Farmacologia, Ribeirão Preto, SP – Brazil

**Keywords:** Nitric Oxide, Nitrites, Exercise, Pulse Wave Analysis, Hypertension

## Abstract

**Background:**

Nitric Oxide (NO) plays an important role in blood pressure (BP) regulation, acting directly on peripheral vascular resistance through vasodilation. Physical training (via eNOS/NO) and intake of nitrite have been considered major stimuli to increase NO.

**Objective:**

We examined the effects of oral nitrite administration and aerobic exercise training on BP and arterial stiffness in Wistar rats.

**Methods:**

Thirty-nine (39) young male Wistar rats were divided into the following groups (n = 9 or 10 per group): Sedentary-Control (SC), Sedentary-Nitrite (SN), Trained-Control (TC), and Trained-Nitrite (TN). They were submitted to aerobic physical training on treadmills for 8 weeks (50-60% of physical capacity, 1h/day, 5 days/week) or kept sedentary. In the last 6 days of training, oral nitrite was administered (15 mg/Kg by gavage). BP, arterial stiffness, and plasma and tissue nitrite concentrations were assessed after the training and oral nitrite administration period. The significant level was defined as p < 0.05.

**Results:**

Oral administration of nitrite was effective in reducing arterial stiffness values (TN, -23%; and SN, -15%). Both groups that had only one type of intervention showed lower systolic BP compared with control (TC vs. SC, -14.23; and SN vs. SC, - 12.46).

**Conclusion:**

We conclude that short-term oral administration for 6 days and an aerobic physical training program promote several hemodynamic benefits in male Wistar rats, such as improvements in arterial stiffness and BP. These responses suggest that physical training and sodium nitrite supplementation can be alternatives for the prevention and treatment of hypertension.

## Introduction

It is well known in scientific literature that blood pressure (BP) should be close to 110-115 mmHg for systolic blood pressure (SBP) and 70-75 mmHg for diastolic blood pressure in healthy individuals. The values above are related to a high incidence of cardiovascular events and mortality, such as stroke and acute myocardial infarction, among others.^1-4^ Several factors are involved in BP regulation, including arterial stiffness (AS) which is characterized by loss of compliance of arterial walls.^5,6^ Currently, due to its consequences, it has been linked as a predictor of cardiovascular mortality risk even in the general population,^7,8^ as stiffer arteries promote less efficient blood supply through the vessels to tissues and organs, placing increasing demands on the heart.^9^

Conversely, physical training is recommended to counteract these effects by preventing HT, decreasing sympathetic tone, and improving peripheral vascular resistance and AS.^10-12^ Physical exercise can improve many mechanisms related to the regulation of BP levels, such as nitric oxide (NO) production and its bioavailability.^13-15^

Considered as a potent vasodilator, NO can be produced through two main metabolic pathways: a) eNOS/NO pathway, the production of NO via eNOS occurs from the cleavage of L-arginine into NO and L-citrulline. In aerobic exercise training, this occurs through physical stimulation due to the increase in cardiac output and the consequent promotion of shear stress in vascular endothelial cells.^16^ This pathway receives a great influence from physical training;^17,18^and b) Nitrate/Nitrite/NO pathway, in which NO concentration is increased through oral administration of nitrite (NO_2_^-^) and nitrate (NO_3_^-^).^19,20^ Initially, the ingested nitrite is converted into NO under acidic conditions in the stomach, while the remainder enters the blood circulation. In the tissues, the remaining nitrite is further reduced under low pH and hypoxic conditions by reductase proteins, converting nitrite to NO, being considered a good source of NO independent of eNOS.^21-23^

As aforementioned, physical exercise and intake of nitrate and nitrite are considered great stimuli to increase NO concentration. Overall, regardless of the production source (eNOS/NO/GMPc or Nitrate/Nitrite/NO pathways), NO plays an important role in controlling BP, acting directly on peripheral vascular resistance through vasodilation.^24^ However, it is still unclear what the effects of combining the two interventions would be on increasing NO concentration and its impact on AS.

Therefore, the purpose of this study was to verify the effects of oral nitrite administration and aerobic exercise training on BP and AS in Wistar rats. In this study, we hypothesize that oral nitrite administration associated with physical training promotes additional effects on BP regulation and AS than isolated interventions.

## Methods

### Animals

Forty Wistar rats (8-week-old / males / 250-300g) were obtained from the Animal breeding Facility of the São Paulo State University (UNESP-Botucatu, SP, Brazil). The animals were housed in the Animal Facility of UNESP-School of Sciences (Bauru, SP, Brazil) in cages of up to five animals in a 12-hour light/dark cycle and controlled temperature (22 °C). All procedures were approved by the Ethics Committee for the Use of Animals (CEUA) of São Paulo State University, Bauru, Brazil (protocol no. 003/2018).

### Experimental groups, physical training, and pharmacological treatment

Initially, all animals underwent a 10-day adaptation period on a treadmill (5-10 m/min, 5 minutes). The animals were submitted to the maximum capacity test (Tmax), which consists of running on a progressive treadmill with increments of 5 m/min every 3 minutes until exhaustion, as previously described. The Tmax was repeated at the end of the 4th week to adjust the speed to maintain training intensity and at the end of the experimental protocol to verify the effects of training on physical capacity.^25,26^After the initial tests, all rats were separated into four groups with similar body weight, pulse wave velocity, and Tmax performance baseline values. Then, these groups were randomly selected to compose the following groups (n=9 or 10 per group): (1) Sedentary-Control (SC), (2) Sedentary-Nitrite (SN), (3) Trained-Control (TC), and (4) Trained-Nitrite (TN). The randomization method chosen was simple randomization. The sample size was based on previous present studies.^27,28^ The training groups performed aerobic physical training on an ergometric treadmill (Inbramed, Millenium, Brazil), with moderate intensity, at 50-60% of Tmax, for 1h/day, 5 days a week, over 8 weeks, while the non-exercised groups remained sedentary. In the last 6 days of training, nitrite was administered.

Sodium nitrite (15 mg/kg of body weight - Dinâmica®) or vehicle (water) was administered by gavage one hour before physical training (at 9 a.m.). The sodium nitrite concentration follows the previously published literature, which demonstrates the antihypertensive and antioxidant effects of the compound.^29-31^

### Pulse Wave Velocity (PWV)

The pulse wave velocity (PWV) method has been used for the assessment of AS and it is considered the gold standard for arterial compliance.^32,33^

To assess the PWV, animals were anesthetized with ketamine hydrochloride and xylazine (50mg/kg and 10 mg/kg, respectively, i.p) and placed in the prone position on a heating table. Two pOpet® sensors (Axelife SAS, Saint Nicolas de Redon, France) were placed on the right forelimb and hindlimb. The traveled distance (D, m) estimated the distance between the two probes and transit time (TT, ms), measured by the pOpet 1.0 software, and was used to calculate the PWV by the following formula: PWV(m/s) = D(m)/TT(s). This method aimed to assess AS and recently demonstrated good validation.^32^ The average of ten measurements was considered as the result. This assessment occurred at two moments: at the beginning of the experiment and the end of the period of physical training and treatment.

### Non-invasive blood pressure assessment

Tail-cuff Plethysmography system was used to determine indirect BP (PanLab LE5001, Barcelona, Spain). All animals were adapted for 5 days to the cylindrical acrylic tube that kept the rats at rest. Animals were kept in a cylindrical acrylic tube, preheated (37 °C) to promote caudal artery vasodilation, and then waited for 5-10 minutes to ensure that the animals were at rest. The cuff was placed in the proximal portion of the tail and connected to the sphygmomanometer to inflate. The measurement was analyzed by the pressure transducer (IITC Inc. Life Science - MRBP-r). The evaluation took place 1 hour after the last nitrite ingestion, and the average of five measurements (1-minute intervals between them) was considered as the result.^33^

### Euthanasia procedures

The animals were euthanized by excess anesthetics, with xylazine hydrochloride (ANASEDAN®, 40mg/kg) and ketamine hydrochloride (DOPALEN®, 160mg/kg), VETEBRANDS Brazil (proportion 1:1, 0.1mg/100g of body weight), and then, decapitation. Euthanasia took place 2 hours after the last nitrite intake. Plasma, cardiac muscle, tibialis anterior, and soleus muscle were collected from the animals.

### Measurement of nitrite concentrations in plasma and tissues

The plasma, cardiac, and muscle tissue (~125mg) were homogenized in a phosphate buffer. After centrifugation, the solution containing the tissues or plasma aliquots was analyzed in duplicate for nitrite concentrations using the ozone-based reductive chemiluminescence method and subsequently evaluated by a gas phase chemiluminescence NO analyzer (Sievers Model 280 analyzer NO; Boulder, CO, USA), as previously described.^20,34^

### Statistical analysis

Descriptive statistics are presented as mean ± standard deviation (SD). The Shapiro-Wilk test was used to test for the normal distribution of the data. A Two-Way ANOVA was used to identify statistical differences in the SBP and plasmatic, muscle, and cardiac nitrite concentrations between groups (SC, SN, TC, and TN). In the presence of interactions, Tukey’s post hoc test was used, and the significant level was defined as p < 0.05.

For PRE and POST comparison in the PWV within groups (SC, SN, TC, and TN), an ANOVA of repeated measures was adopted. The data were analyzed using the SigmaPlot 12.0 statistical package (Systac Software, Inc., San Jose, CA, USA).

## Results

In the central image, we present the main findings of our study. All the groups showed similar results for PWV at the start of the study, demonstrating the homogeneity of the groups in this regard. Both treated groups had lower PWV values compared to the untreated groups (-19% SN vs. SC; -24% TN vs. TC). Figure 1 also shows the comparison between the pre and post-moments. No significant differences were found for the SC and TC groups for PWV. However, the TN (-23%) and SN (-15%) groups showed significant reductions in PWV after the training or treatment period.

**Figure 1:** – Pulse wave velocity (PWV) before (pre) and after (post) in SC (Sedentary-Control, n=10), SN (Sedentary-Nitrite, n=10), TC (Trained-Control, n=10), and TN (Trained-Nitrite, n=9). *Statistical differences vs. respective control and - (SN vs. SC, p=0.003; and TN vs. TC, p=<0.001); # statistical differences between PRE and POST-intervention – (TN, p=0.007; SN, 0.011); level of significance p<0.05.

Figure 2 presents lower SBP in SN and TC compared to the SC group. This difference reached 12.46 mmHg in the SN group (p=<0.001) and 14.23 mmHg in the TC group (p=<0.001). However, the combination of physical training and oral administration of nitrite did not present additional benefits to those already found in isolation.

**Figure 2:** – Systolic blood pressure (SBP) values in SC (Sedentary-Control, n=10), SN (Sedentary-Nitrite, n=10), TC (Trained-Control, n=10), and TN (Trained-Nitrite, n=9). *Statistical differences, TC vs SC, p=<0.001; SN vs. SC, p=<0.001; level of significance p<0.05.

Figure 3 presents the nitrite concentrations in the different tissues. It can be observed that both treated groups (SN and TN) presented higher values compared with non-treated groups (SC and TC) in plasma (Figure 3A), heart (Figure 3B) and soleus (Figure 3C), except for the tibialis anterior muscle (Figure 3D).

**Figure 3:** – Nitrite concentrations in plasma nitrite (A), heart (B), and soleus skeletal muscle (C) and anterior tibial skeletal muscle (D) in SC (Sedentary-Control, n=10), SN (Sedentary-Nitrite, n=10), TC (Trained-Control, n=10), and TN (Trained-Nitrite, n=9). *Statistical differences, Plasm – SN vs. SC, p=0.006; TN vs. TC, p=<0.001; Heart – SN vs. SC, p=0.002; TN vs. TC, p=<0.001; Soleus – SN vs. SC, p=0.005; TN vs. TC, p=0.003; Tibialis Anterior – SN vs. SC, p=0.019; level of significance p<0.05.

No statistical difference was found between the SC and TC groups for plasma nitrite concentration. However, nitrite treatment induced an increase in the SN group (p=0.006) and TN group (p=<0.001) compared with their respective control groups.

The magnitude of the increase in cardiac and skeletal muscle (soleus) nitrite concentration was smaller compared with plasmatic nitrite concentration; however, the same tendency was found. No difference was found between the SC and TC groups for cardiac nitrite concentration. However, nitrite treatment induced an increase in the SN group (by approximately four times) and TN group (by approximately eight times) compared with their respective control groups.

For muscle tissue, no difference was found between SC and TC groups in soleus nitrite concentration and anterior tibial nitrite concentration. Moreover, the treated groups (SN and TN) showed differences concerning their respective controls in the soleus muscle (both by approximately four times). However, in the tibialis anterior muscle, only the SN group showed a difference compared to its control (by approximately three times).

## Discussion

The purpose of this study was to verify the effect of oral nitrite administration on BP and AS in trained and sedentary rats. Briefly, our results demonstrated that the oral nitrite administration for a short period (6 days) was related to a 15% and 22.1% reduction in the PWV in the SN and TN, respectively, compared with groups that did not receive pharmacological treatment.

AS has been extensively studied in experimental animal models,^5,6,32,35^ and it is considered an important predictor of cardiovascular risk in the general population.^7^ Although the group that underwent only physical training did not present a reduction in PWV, there is some evidence in the literature that physical exercise promotes benefits on AS. In a systematic review by Lopes et al.,^35^ it was demonstrated that aerobic training, combined training, and resistance training can promote a reduction in PWV in hypertensive patients.

Nitrite concentration was higher in both groups that received oral nitrite supplementation, which was expected due to the study design. However, these results may contribute to improvements in the vascular endothelium and improvements in NO bioavailability. As a consequence, it contributes to the antihypertensive effects.^18^ It has been demonstrated that nitrite supplementation protects the vascular endothelium from antioxidant activity, reducing/inhibiting the activity of NADPH oxidase and xanthine oxidoreductase (XOR) in animals,^36,37^ not evaluated by this study. The inhibition of NADPH oxidase activity is an important mechanism since it is the most prominent source of reactive oxygen species (ROS), and it works by inactivating NO.^38-40^ Ling et al.^18^ also demonstrated that oral nitrite administration promotes positive changes in NADPH oxidase expression.

In addition, oral administration of nitrite also proved to be effective in increasing nitrite concentrations in plasma and other tissues (cardiac muscle and soleus), with higher values being found in the treated groups compared to control groups. These results agree with the literature, which reports that nitrite supplementation increases nitrite concentrations in plasma and other tissues.^18,30,41^ Conversely, in the tibialis anterior muscle, only the sedentary group that received treatment showed high nitrite concentration values. It should also be noted that the literature has shown that there are changes in plasma and tissue nitrite concentrations over time after oral administration.^20^ Pinheiro et al. showed that the increase in nitrite concentrations in plasma began 15 minutes after treatment and remained high for up to 4 hours. In the heart and skeletal muscle, concentrations remained high up to 2 hours after treatment, thus demonstrating the relationship between concentration and treatment time. Therefore, this relationship confirms our hypothesis that oral administration of sodium nitrite may be an alternative for increasing nitrite concentrations and, consequently, possibly NO bioavailability.

According to the BP results, both interventions, when carried out in isolation, whether physical training or pharmacological treatment, showed a reduction in SBP compared to the sedentary and untreated group. Although many studies have shown that aerobic exercise training does not reduce BP levels in Wistar animals.^42,43^ In our study, although the animals used in this study were considered normotensive (the strain of animals was normotensive), it can be seen in the SC group that the SBP values were around 140 mmHg, which showed altered BP values. Because of this, treatment with nitrite or physical training were sufficient to normalize BP values. A possible explanation for these results is the increase in NO production and concentration, the decrease in ROS concentration, increase in vascular endothelial growth factor (VEGF), which are responsible for the activation of the angiogenesis mechanism, reduction of Ang-II production, and lower sympathetic activity.^10,42,44-46^ In addition, nitrite supplementation has demonstrated benefits in vascular relaxation,^47^ and improvements in cardiac function and remodeling.^48^

The limitations of this study include the short period of treatment with nitrite, which requires further studies to verify the long-term response. The SBP levels of animals were close to 140 mmHg, which is considered an altered level for Wistar animals, and yet we were only able to assess BP at the end of the protocol. So, we can’t say that the physical training and treatment reduced SBP throughout the program.

## Conclusion

In summary, short-term (6 days) oral administration or an aerobic physical training program promotes several hemodynamic benefits in male Wistar rats, such as improvements in AS and BP. These responses suggest that physical training and sodium nitrite supplementation can be alternatives for the prevention and treatment of hypertension.
